# Stressful Life Events, Sleep Quality and Non-Suicidal Self-Injury in Chinese Adolescents: The Moderating Effect of Sensation Seeking

**DOI:** 10.3390/bs14040286

**Published:** 2024-03-30

**Authors:** Yuanxiu Ye, Huahua Wang, Xiaoyan Liao, Chengfu Yu, Wei Zhang

**Affiliations:** 1 School of Psychology, South China Normal University, Guangzhou 510631, China; 2 Department of Psychology, Research Center of Adolescent Psychology and Behavior, School of Education, Guangzhou University, Guangzhou 510006, China

**Keywords:** stressful life events, sleep quality, sensation seeking, non-suicidal self-injury (NSSI), adolescents

## Abstract

Despite the growing evidence that stressful life events are associated with adolescent non-suicidal self-injury (NSSI), few studies have investigated the mediating and moderating mechanisms underlying this link. The current study examined whether sleep quality mediated the link between stressful life events and NSSI and whether this mediating process was moderated by adolescent sensation seeking. The participants were 1006 Chinese adolescents (48.21% boys; *mean age* = 13.16 years, *SD* = 0.67). They completed the Life Events Checklist, Pittsburgh Sleep Quality Index, UPPS-P Impulsive Behavior Scale, and NSSI Questionnaire. The PROCESS macro for SPSS was used to examine the hypothesized moderated mediation model. The results showed that sleep quality significantly mediated the positive association between stressful life events and adolescent NSSI. Moreover, this mediating pathway was moderated by sensation seeking. Specifically, the risk impact of stressful life events on NSSI through sleep quality was significant only among adolescents with high-level sensation seeking but not among those with low-level sensation seeking. These findings provide intervention insights to reduce the risk of adolescent NSSI.

## 1. Introduction

Non-suicidal self-injury (NSSI) refers to inflicting direct and purposeful harm to one’s own body without suicidal intention [1]. NSSI involves a variety of behavioral patterns, such as cutting, burning, hitting, scratching, and hair pulling, with cutting or carving of the skin being the most common method, usually used as coping mechanisms by individuals experiencing emotional distress [2]. This behavior typically emerges in early adolescence and is prevalent across various gender identities, ethnic backgrounds, and socioeconomic statuses [1]. Previous research shows that adolescents have the highest prevalence of NSSI compared to other age groups, with rates of 17.2% among adolescents, 13.4% among young adults, and 5.5% among adults [3]. The high prevalence of NSSI in adolescents emphasizes the importance of gaining a deeper understanding of NSSI within this age group. A recent study reported that the prevalence of NSSI in Chinese adolescents was 16.4% over the past six months [4]. Early adolescent NSSI not only predicts later psychiatric disorders such as depression, anxiety, and eating disorders but also increases the risk of suicidal thoughts and attempts among adolescents [5,6]. Therefore, further research is needed on the risk factors and underlying causes of adolescent NSSI to identify targeted interventions.

Stressful life events are a sequence of adversities or challenges that individuals can experience in their daily lives [7]. The integrated theoretical model of NSSI suggests that stressful life events are significant triggers for NSSI [8]. Consistent evidence also indicates that adolescents engage in NSSI more frequently after experiencing stressful life events [9,10,11]. For example, a prospective study of 529 adolescents found that stressful life events significantly predicted adolescent NSSI one year later [12]. However, the mediating and moderating mechanisms underlying the relationship between stressful life events and adolescent NSSI remain largely unknown. Therefore, this study aimed to investigate whether the relationship between stressful life events and NSSI in adolescents is mediated by sleep quality. If true, the study also aimed to determine whether this indirect link is moderated by sensation seeking, a personality characteristic that reflects an inclination toward pursuing new and thrilling experiences [13].

Researchers have recognized that sleep problems are common among Chinese adolescents, with 26.7% experiencing such issues [14]. However, few studies have explored the antecedents and consequences of sleep quality simultaneously in one model. According to the stress process theory [15], exposure to stressful life events can overwhelm individuals, both mentally and physically (e.g., through poor sleep quality), which will increase the likelihood of the emergence of various problems (e.g., NSSI). This theory specifies that stressful life events can be an antecedent to poor sleep quality, of which NSSI is a possible consequence. That is, sleep quality may act as a mediator between stressful life events and NSSI.

Stressful life events can trigger physiological responses such as heightened activation of the sympathetic nervous system, ultimately leading to the disruption of sleep quality [16]. The association between experiencing stressful life events and poor sleep quality has been consistently observed in studies involving both Chinese and Western adolescents [14,17]. Poor sleep quality may subsequently increase the risk of NSSI. In particular, poor sleep quality may damage adolescent emotional regulation, increase the risk of depression, and further increase the risk of NSSI [18]. Multiple studies have demonstrated that poor sleep quality significantly predicts adolescent NSSI [19,20,21]. Moreover, some studies have indicated that sleep quality acts as a mediator in the relationship between stressful experiences and suicidality [22,23]. In a study conducted by King et al. [23], the fear of sleep and sleep quality played a significant sequential mediating role in the association between childhood trauma and likelihood of suicidal attempt. Specifically, the findings indicated that childhood trauma contributed to an increased fear of sleep, which subsequently negatively impacted sleep quality, ultimately increasing the risk of suicidal attempt. Drawing insights from the existing theoretical framework and empirical findings, it is plausible to hypothesize that sleep quality mediates the relationship between stressful life events and adolescent NSSI.

The available evidence indicates a connection between stressful life events, sleep quality, and adolescent NSSI [10,21]. Nonetheless, not every adolescent facing stressful life events and experiencing poor sleep quality will resort to NSSI. Hence, both protective and risky factors are assumed to influence the strength of this link. In this study, we focus on sensation seeking as a potential risk factor that could elucidate variations in how adolescents respond to stressful life events and inadequate sleep quality. Specifically, we examine the role of sensation seeking as a moderator in the direct and indirect association between stressful life events and adolescent NSSI.

The biosocial-affect model of sensation seeking posits that high-level sensation seekers are prone to engaging in risky behaviors because of their emotional attraction to such behaviors and low risk perception [24]. According to this model, adolescents with high-level sensation seeking may be more attracted to NSSI and have higher rates of NSSI engagement when they experience stressful life events than do adolescents with low-level sensation seeking. In other words, the direct and indirect effects of stressful life events on NSSI may vary with adolescent sensation seeking. Previous studies have revealed the predictive ability of sensation seeking in determining the frequency of NSSI and the diversity of NSSI methods in adolescents [25].

In addition, previous research has suggested that sensation seeking exacerbates the effect of external risk factors on emotional and brain activation. For instance, one study found that, when exposed to external stimuli, individuals with high-level sensation seeking have higher emotional activation and response than those with low levels [26]. Furthermore, some functional magnetic resonance imaging (fMRI) studies have demonstrated that, compared to individuals with low-level sensation seeking, those with high-level sensation seeking display greater brain and neural activation in response to risk cues [27,28]. As mentioned above, high physiological activation affects sleep quality [16]. These collective findings suggest that sensation seeking could potentially moderate the association between external risk factors and sleep quality in adolescents. Interestingly, while previous research has explored the interaction between sensation seeking and external risk factors, particularly in relation to emotional and brain activation, there remains a gap in the literature regarding whether sensation seeking moderates the pathway from stressful life events (as an external risk factor) to sleep quality in adolescents.

Sensation seeking may also play a moderating role in the relationship between sleep quality and adolescent NSSI. Earlier studies have revealed that the interplay between sensation seeking and sleep quality is associated with an elevated risk of antisocial behavior in the youth [29]. Moreover, research has provided empirical evidence that high-level sensation seeking can amplify the adverse effects of sleep problems on adolescent risk behaviors [30]. Specifically, Rusnac et al. [30] found that, compared to those with normal sleep patterns, sleep-deprived youth with high-level sensation seeking demonstrated more risky behaviors, whereas sleep-deprived youth with low-level sensation seeking did not show the same tendency. Thus, adolescent sensation seeking may also moderate the pathway from sleep quality to NSSI.

In summary, this study aimed to examine the mediating effect of sleep quality and the moderating effect of sensation seeking in the pathway from stressful life events to NSSI in Chinese adolescents. Based on the literature review, this study proposed the following hypotheses:

**H1.**  *Sleep quality mediates the relationship between stressful life events and adolescent NSSI.*

**H2.**  *Sensation seeking moderates the indirect relationship between stressful life events and adolescent NSSI via sleep quality.*

**Figure 1:**
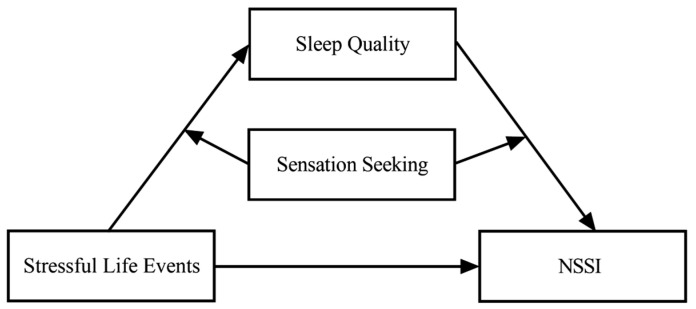
Hypothetical moderated mediation model. Note: NSSI, non-suicidal self-injury.

Figure 1 illustrates the hypothetical moderated mediation model.

## 2. Materials and Methods

### 2.1. Participants

In this study, participants were selected using a stratified and random cluster sampling approach. Specifically, 18 classes were randomly chosen from three junior high schools in Guangdong Province, China. Each school selected six classes randomly, consisting of three from Grade 7 and three from Grade 8. Grade 9 students were not part of the survey due to the academic stress they were under. Following the exclusion of participants who did not complete the questionnaire seriously, 1006 participants completed the survey attentively for this study. Participants were aged 12–15 years, with an average age of 13.16 years (*SD* = 0.67 years), and 485 participants (48.21%) were boys. Approximately half of the participants (55.49%) were from urban areas. In addition, 48.63% of participants’ fathers and 54.07% of their mothers had less than a high school education.

### 2.2. Procedure

Before data collection, the researchers obtained approval from the Academic Ethics Review Board of Guangzhou University (GZHU 2019012) and informed consent from both participants and their parents. The informed consent emphasized that participation was completely voluntary and that data from the questionnaires were confidential and only obtained for research purposes. All questionnaires were completed in the students’ original classrooms. Two trained research assistants were available in each classroom to guide the students as they completed the questionnaires. The entire data collection process lasted approximately 30 min. As an incentive, participants were rewarded with a signature pen upon completing the questionnaire survey.

### 2.3. Measures

#### 2.3.1. Stressful Life Events

The Chinese version of the Adolescent Self-Rating Life Events Checklist [7] was used to measure stressful life events. This scale has shown excellent reliability and validity in various studies conducted with Chinese participants [10,14]. The scale contains 27 items (e.g., being discriminated against and treated coldly by others). Participants were asked to determine whether these 27 events occurred in the past six months. If an event did not occur, they would rate it as 0. If an event did occur, participants were asked to rate the level of distress caused by that event on a five-point scale (1 = “no distress” to 5 = “extreme distress”). Higher average scores of all items indicated greater exposure to stressful life events. The Cronbach’s α was 0.92 for this scale in this study.

#### 2.3.2. Sleep Quality

The sleep quality of participants in the previous month was measured by the Chinese version [31] of the Pittsburgh Sleep Quality Index [32]. The scale has been widely employed in research involving Chinese populations, consistently demonstrating strong reliability and validity [14,21]. The scale comprises 18 items (e.g., waking up easily at night) across 7 subscales. All items were scored on a four-point scale (0 = “none” to 3 = “average of three or more nights per week”). The score for each subscale was calculated separately; then, the average score of the subscales was determined. Higher scores indicated poor sleep quality. The Cronbach’s α was 0.77 for this scale in this study.

#### 2.3.3. Sensation Seeking

The Chinese version [33] of the sensation-seeking subscale of the UPPS-P Impulsive Behavior Scale [34] was used to measure participants’ levels of sensation seeking. Previous research has shown that this scale exhibits satisfactory reliability when applied to Chinese adolescents [33,35]. The scale included four items related to sensation seeking (e.g., “I sometimes like doing things that are a bit frightening”). Items were scored from 1 (“strongly disagree”) to 4 (“strongly agree”) and then the scores were averaged. Higher scores indicated higher levels of sensation seeking. The Cronbach’s α was 0.74 for this scale in this study.

#### 2.3.4. Non-Suicidal Self-Injury

The Chinese version of the Adolescent NSSI Questionnaire [36] was used to assess participants’ NSSI in the past six months. The questionnaire has been utilized in previous research to measure NSSI among Chinese adolescents, showing good reliability and validity [35,37]. The questionnaire includes seven NSSI behaviors (e.g., “self-cutting, burning, and scratching skin”), all scored on a four-point scale (1 = “never” to 4 = “six times or more”). We used the mean score of the questionnaire to represent the frequency of NSSI for each participant. A higher score indicated a higher frequency of NSSI. The Cronbach’s α was 0.71 for this scale in this study.

#### 2.3.5. Rejection Sensitivity

Adolescent rejection sensitivity was measured by the Chinese version [38] of the Rejection Sensitivity Questionnaire [39]. This scale is a widely used measure with good reliability and validity in rejection sensitivity evaluation among Chinese adolescents [40,41]. This scale contains 18 items (e.g., “I’ve always been afraid of letting people down”), all scored on a five-point scale (1 = “not at all true” to 5 = “always true”). The mean score of all items was used to represent the rejection sensitivity of each participant. A higher score indicated a higher level of rejection sensitivity. The Cronbach’s α was 0.86 for this scale in this study.

### 2.4. Data Analyses

Data were analyzed using SPSS 25.0. The analyses involved several steps. First, descriptive and bivariate correlation analyses were performed. Second, SPSS macro PROCESS Model 4 [42] was used to examine whether sleep quality mediated the association between stressful life events and NSSI. Third, SPSS macro PROCESS Model 58 [42] was used to further test the moderating effect of sensation seeking on the indirect association between stressful life events and NSSI via sleep quality. Bootstrapping (*n* = 5000) was performed to estimate the 95% bias-corrected confidence intervals (95% CI). If the 95% CI did not contain zero, the moderated mediating model was supported. According to previous research [43,44], gender, age, and rejection sensitivity are significantly associated with NSSI. Thus, the present study incorporated gender, age, and rejection sensitivity as covariates in the analysis.

## 3. Results

### 3.1. Descriptive Statistics

Table 1 presents the means, standard deviations, and correlations of all the study variables. As shown in Table 1, stressful life events, NSSI, sleep quality, and sensation seeking are significantly positively correlated with each other.

### 3.2. Mediating Effect of Sleep Quality

**Figure 2:**
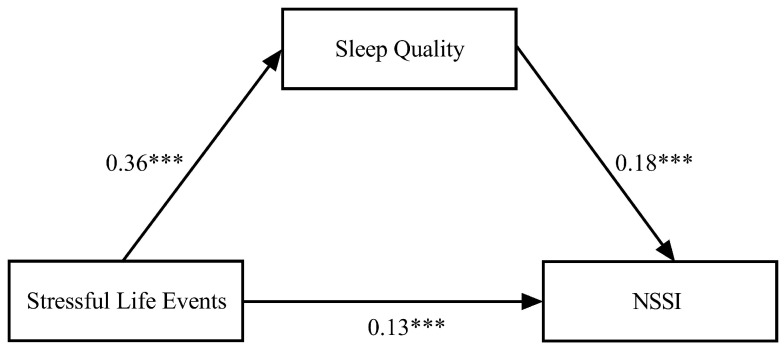
The mediating effect of sleep quality in the relationship between stressful life events and NSSI. Note: NSSI, non-suicidal self-injury. *** *p* < 0.001.

After controlling for covariates, the results (see Figure 2) showed that stressful life events significantly predicted sleep quality (*β* = 0.36, *p* < 0.001). Furthermore, sleep quality significantly predicted NSSI (*β* = 0.18, *p* < 0.001). Moreover, stressful life events significantly predict NSSI (*β* = 0.13, *p* < 0.001). Thus, sleep quality partially mediated the relationship between stressful life events and NSSI. Bootstrapping analyses (*n* = 5000) showed that the mediating effect of sleep quality was statistically significant (*β* = 0.06, *SE* = 0.02, 95% CI [0.04, 0.10]). The contribution of the indirect effect to the total effect was 31.58% (see Table 2).

### 3.3. Moderated Mediation Model

**Figure 3:**
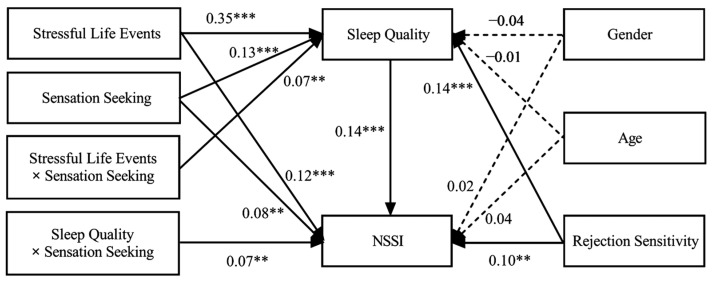
The moderated mediation model. Note: NSSI, non-suicidal self-injury. Gender coded as male = 1, female = 0. The numbers presented on the graph are standardized regression coefficients. Dotted lines represent non-significant pathways. Gender, age, and rejection sensitivity are included as covariates. ** *p* < 0.01, *** *p* < 0.001.

**Figure 4:**
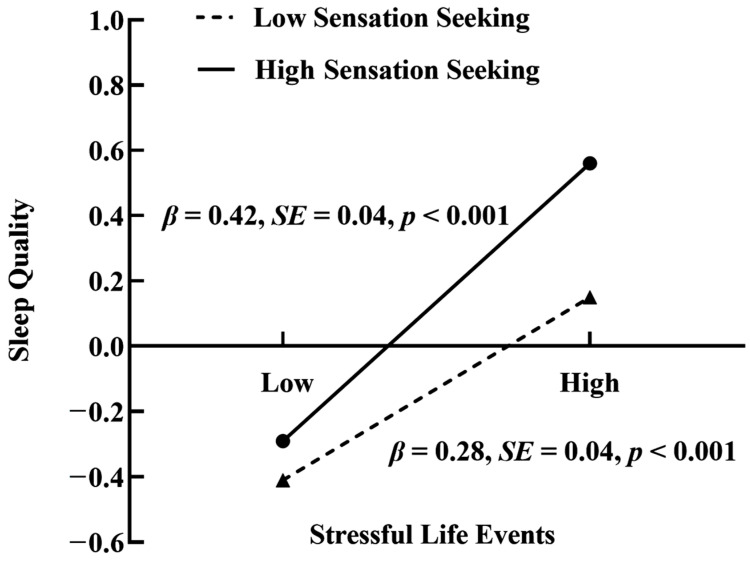
Interactive effect of stressful life events and sensation seeking on sleep quality.

**Figure 5:**
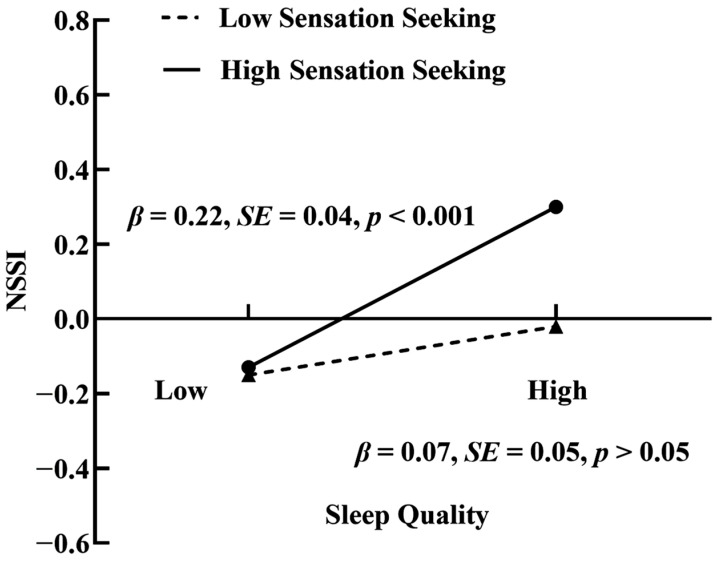
Interactive effect of sleep quality and sensation seeking on NSSI. Note: NSSI, non-suicidal self-injury.

When controlling for covariates, the results (see Figure 3) showed that stressful life events interacted with sensation seeking to predict sleep quality (*β* = 0.07, *p* < 0.01), and sleep quality interacted with sensation seeking to predict NSSI (*β* = 0.07, *p* < 0.01). A simple slope test was then performed to better understand the moderating role of sensation seeking. As shown in Figure 4, stressful life events more strongly predicted sleep quality among participants with high-level sensation seeking (*β* = 0.42, *p* < 0.001) than those with low-level sensation seeking (*β* = 0.28, *p* < 0.001). As shown in Figure 5, for participants with high-level sensation seeking, sleep quality positively predicted NSSI (*β* = 0.22, *p* < 0.001); however, for participants with low-level sensation seeking, no such predictive effect was found (*β* = 0.07, *p* > 0.05).

We further examined whether the mediating effect of stressful life events on NSSI via sleep quality was conditioned by sensation seeking. The bias-corrected percentile bootstrap results (see Table 3) showed that the mediating effect of sleep quality was statistically significant only when sensation seeking was high (*β* = 0.09, *SE* = 0.03, 95% CI [0.05, 0.15]) but not when sensation seeking was low (*β* = 0.02, *SE* = 0.02, 95% CI [−0.01, 0.06]).

## 4. Discussion

Consistent with H1, this study found that the relationship between stressful life events and adolescent NSSI is mediated by sleep quality. Earlier studies indicated that sleep quality is independently associated with both stressful life events [14] and adolescent NSSI [21]. This study expanded on previous research by revealing an unfolding pathway from stressful life events to sleep quality to NSSI in adolescents. These results suggest that experiencing various stressful life events significantly damages the sleep quality of adolescents, subsequently increasing their risk of engaging in NSSI. Stressful life events can induce stress, resulting in increased activation of the locus coeruleus norepinephrine system and hypothalamic–pituitary–adrenal axis [45]. This heightened activation can lead to increased arousal and compromised sleep quality [45]. Furthermore, adolescents with poor sleep quality often struggle with impulse control, contributing to an escalation in NSSI [46]. These findings support the stress process theory [15].

Supporting H2, sensation seeking was found to be a moderator of the indirect pathway of stressful life events to NSSI. Specifically, stressful life events significantly predicted an increase in NSSI via poor sleep quality but only for adolescents with high-level sensation seeking. These findings suggest that high-level sensation seeking amplifies poor sleep quality among adolescents who have experienced various stressful life events. Individuals who exhibit high-level sensation seeking may face challenges in effectively managing impulsive emotions triggered by stressful life events, which could potentially lead to disrupted sleep patterns and poor sleep quality [47]. Moreover, the relationship between sleep quality and NSSI was amplified by high-level sensation seeking. Sleep can help individuals recover from long-lasting adverse consequences of stressful events, whereas poor sleep quality results in reduced self-control [48], diminishing the inhibitory effect that restrains adolescents from engaging in NSSI. Meanwhile, adolescents with high-level sensation seeking actively pursue novel stimuli, intensifying the motivation toward NSSI [13,35]. Consequently, the combination of poor sleep quality and elevated sensation seeking increases the risk that adolescents will engage in NSSI. These results are congruent with the biosocial-affect model of sensation seeking [24] and provide further evidence that high levels of sensation seeking magnify the indirect mechanism through which stressful life events lead to NSSI.

This is the first study designed to examine the factors influencing adolescent NSSI by integrating stressful life events, sleep quality, and sensation seeking. The findings not only contribute to the synthesis and expansion of existing theories but also have significant implications for future practical applications. First, this study identified that sleep quality acts as a mediator between stressful life events and NSSI in adolescents, highlighting the importance of addressing sleep quality in interventions for adolescent NSSI. Therefore, ensuring that adolescents obtain sufficient sleep is crucial. If they experience persistent or severe sleep problems, sleep counseling and guidance may be effective in improving sleep quality. Second, this study found that the mediating effect of sleep quality in the relationship between stressful life events and adolescent NSSI was significant only among adolescents with high-level sensation seeking. This finding suggests that adolescents with high-level sensation seeking are more susceptible to stressful life events and more likely to develop poor sleep quality and engage in NSSI than their low-level sensation-seeking counterparts. Thus, more attention should be paid to adolescents who display high levels of sensation seeking, who may benefit more from programs that aim to enhance strategies for coping with stressful events and improve sleep quality.

This study had several limitations. First, all measures were self-reported, which may have introduced the respondent recall bias. Future research could employ various tools to collect data and obtain a more impartial perspective. Second, this study focused only on sensation seeking (a risk factor), which amplified the indirect link between stressful life events and NSSI. To enhance the understanding of this relationship, future research should validate the protective factors that can mitigate the association between stressful life events and NSSI. Third, the study used a sample that consisted solely of Chinese nonclinical participants, which limited its generalizability. Future research should aim for a more diverse sample with respect to race/ethnicity and clinical status to enhance the external validity of the findings. Finally, a cross-sectional design limited the ability to explore the dynamics between variables. Future studies could use multiple waves of longitudinal data to reveal the dynamics and complex relationships between variables over time.

## 5. Conclusions

This study advances the existing literature by revealing the explanatory path linking stressful life events with NSSI and examining whether this path varies according to adolescent characteristics. The results provide evidence that sleep quality has a mediating role in explaining the association between stressful life events and NSSI. Moreover, this mediation is observed only in adolescents who exhibit high-level sensation seeking. These findings can inform interventions that aim to reduce the risk of adolescent NSSI. Implementing comprehensive programs that simultaneously address stressful life events (external factors), sleep quality (internal factors), and sensation seeking (personality characteristics) may be effective in reducing the risk of NSSI in adolescents. It is essential to emphasize that, due to this study employing a one-time survey, the lack of temporal information precludes the determination of the temporal order between sleep quality and NSSI. Consequently, it is plausible that the effect of sleep quality exhibits a synergistic effect in conjunction with other yet-to-be-defined variables, including family structure and factors fostering resilience.

## Figures and Tables

**Table 1:** Descriptive statistics and correlations for the variables.

Variable	1	2	3	4	5	6	7
1. Gender	1.00						
2. Age	0.06 *	1.00					
3. Rejection sensitivity	−0.20 ***	0.01	1.00				
4. Stressful life events	−0.12 ***	0.05	0.33 ***	1.00			
5. Sleep quality	−0.09 **	0.00	0.27 ***	0.41 ***	1.00		
6. NSSI	−0.03	0.04	0.19 ***	0.23 ***	0.25 ***	1.00	
7. Sensation seeking	0.05	−0.06	0.09 **	0.09 **	0.18 ***	0.14 ***	1.00
*Mean*	-	13.16	3.04	1.01	0.52	1.09	2.00
*SD*	-	0.67	0.43	1.02	0.33	0.29	0.67

Note: NSSI, non-suicidal self-injury. Gender coded as male = 1, female = 0. * *p* < 0.05, ** *p* < 0.01, *** *p* < 0.001.

**Table 2:** Total, direct, and indirect effects of stressful life events on NSSI.

	Effect	Boot SE	Boot LLCI	Boot ULCI	Ratio
Indirect effect	0.06	0.02	0.04	0.10	31.58%
Direct effect	0.13	0.03	0.06	0.19	68.42%
Total effect	0.19	0.03	0.13	0.25	100.00%

Note: NSSI, non-suicidal self-injury.

**Table 3:** Mediating effect test results under different sensation seeking levels.

	Effect	Boot SE	Boot LLCI	Boot ULCI
high levels of sensation seeking	0.09	0.03	0.05	0.15
low levels of sensation seeking	0.02	0.02	−0.01	0.06

## Data Availability

The datasets used and analyzed during the current study are available from the corresponding author on reasonable request.
